# Enhanced urine refractive index sensing using a defect-engineered one-dimensional photonic crystal

**DOI:** 10.1038/s41598-026-58385-2

**Published:** 2026-06-25

**Authors:** Arafa H. Aly

**Affiliations:** TH-PPM Group, Physics Department, Faculty of Science, Beni-Suef University, Beni Suef, 62111 Egypt

**Keywords:** Photonic crystal sensor, Defect mode resonance, Urine sensing, Refractive index detection, Optical biosensor, Light–matter interaction, Optics and photonics, Physics

## Abstract

In this work, a defect-engineered one-dimensional photonic crystal (1D PhC) sensor is proposed for high-resolution urine refractive-index detection. The structure consists of alternating TiO₂/MgF₂ layers forming Bragg mirrors with a central urine-filled defect cavity, where a sharp localized resonance is generated inside the photonic band gap. Numerical analysis based on the transfer matrix method shows a stable red shift of the defect mode as the urine refractive index increases from 1.333 to 1.360. The proposed sensor achieves a sensitivity of 388.57 nm/RIU with excellent linearity $\:({R}^{2}=0.999989)$, together with an average FWHM of 0.0305 nm, an average Q-factor of $\:2.55\times\:{10}^{4},$ and an average FOM of $\:1.32\times\:{10}^{2}RI{U}^{-1}$. A tolerance analysis under ± 2.5% thickness variation confirms that the resonance remains narrow and well defined, demonstrating good fabrication robustness. The results also highlight an important principle in photonic sensing: sensor performance should not be judged by sensitivity alone, but by a balanced combination of sensitivity, linewidth, resonance quality, figure of merit, and tolerance against structural deviations. These findings indicate that the proposed design is a promising candidate for practical urine-based biomedical sensing applications. A comparison with representative previously reported photonic-crystal sensing platforms further confirms the balanced overall performance of the proposed design.

## Introduction

Photonic crystals (PhCs) have emerged as versatile platforms for controlling electromagnetic-wave propagation owing to their periodic dielectric architecture, which gives rise to photonic band gaps (PBGs) where light transmission is strongly suppressed^1^. Introducing a structural defect into such periodic systems creates a localized resonant mode within the band gap, leading to strong optical confinement and enhanced light–matter interaction^2^. These characteristics make defect-engineered PhCs highly attractive for advanced optical sensing applications.

In recent years, PhC-based biosensors have attracted considerable attention because of their compactness, label-free operation, and potential for real-time detection^3–6^. Their sensing principle commonly relies on monitoring changes in optical characteristics, such as resonance wavelength, transmission response, or spectral linewidth, induced by refractive-index variations in the surrounding medium^7,8^. Such capabilities have made photonic sensors promising candidates for a wide range of biomedical and biochemical applications. Importantly, however, sensing performance should not be assessed on the basis of sensitivity alone. A meaningful evaluation requires the combined consideration of sensitivity, linewidth, Q-factor, figure of merit, resonance stability, and fabrication tolerance, since these parameters collectively determine the practical efficiency and reliability of the sensing platform.

Among the various photonic crystal architectures, one-dimensional (1D) photonic crystals remain particularly attractive because of their simple geometry, ease of fabrication, and strong light confinement capability^9^. Introducing a defect layer into a periodic 1D structure gives rise to a localized resonance within the photonic band gap, producing a sharp defect mode that is highly sensitive to refractive-index changes in the analyte region^10–13^. Owing to this feature, defect-engineered 1D photonic crystals have become an effective platform for refractive-index sensing.

Recent developments in photonic crystal biosensors have shown that sensor performance can be enhanced substantially through structural optimization and proper material selection. Graphene-based, MoS₂-assisted, and other defect-engineered photonic platforms have demonstrated promising results for the detection of biological analytes, biomolecules, and medically relevant samples^14–17^. These studies indicate that improving refractive-index contrast, strengthening field localization, and carefully tailoring defect-cavity properties can all contribute to better sensing behavior. At the same time, current photonic sensing research increasingly emphasizes that sensor performance should not be evaluated on the basis of sensitivity alone. Although resonance shift is an important metric, the practical quality of a sensor is also determined by other key parameters, such as the full width at half maximum (FWHM), quality factor (Q-factor), figure of merit (FOM), resonance stability, and tolerance to fabrication variations^18,19^. In many cases, a higher sensitivity may be accompanied by linewidth broadening or reduced resonance quality. Therefore, a reliable assessment of photonic sensors requires a balanced multi-parameter evaluation rather than a single sensitivity-based comparison. In this context, the present work focuses not only on achieving a reasonable and competitive sensitivity, but also on maintaining narrow spectral linewidth, high Q-factor, strong defect-mode confinement, and acceptable structural robustness.

Urine analysis is widely recognized as a convenient and non-invasive approach for monitoring a variety of physiological and pathological conditions, since variations in urine composition are directly reflected in its refractive index^18^. This makes urine an attractive target for optical sensing platforms, particularly those capable of detecting subtle refractive-index changes with high precision and fast response^20–23^. In the present study, the urine refractive index was varied from *n* =1.333 to 1.360, covering representative urine optical-property variations associated with differences in urine concentration and composition. In parallel, recent progress in photonic biosensing, including integrated photonic systems and data-assisted sensing strategies, has further expanded the possibilities for improving device performance and supporting real-time biomedical diagnostics^22–25^. These advances continue to reinforce the importance of photonic crystal sensors in next-generation healthcare applications. In this study, a defect-engineered one-dimensional photonic crystal sensor is proposed for urine refractive-index detection. The structure is composed of alternating TiO₂ and MgF₂ layers forming Bragg mirrors, with a central cavity filled with the urine analyte. The sensing mechanism is based on tracking the shift of the localized defect resonance inside the photonic band gap as the refractive index changes. Over the investigated range from 1.333 to 1.360, the proposed design exhibits a stable red shift and achieves a sensitivity of 388.57 nm/RIU with excellent linearity. Beyond sensitivity, the sensor also maintains a very narrow average linewidth, a high Q-factor, and a large FOM, indicating strong spectral selectivity and high resonance quality. The field-distribution analysis further confirms strong electromagnetic confinement inside the defect region, while the tolerance study demonstrates acceptable robustness against moderate fabrication deviations. Taken together, these features show that the proposed platform is a promising candidate for reliable urine-based biomedical sensing, especially when performance is judged through a balanced multi-parameter perspective rather than sensitivity alone.

## Theoretical model and numerical simulation

The proposed device is a one-dimensional photonic crystal consisting of alternating $\:Ti{O}_{2}$ and $\:Mg{F}_{2}$ layers arranged as two symmetric Bragg reflectors, with a defect cavity at the center containing the urine sample. Its optical behavior is investigated through the transfer matrix method $\:\left(\mathrm{T}\mathrm{M}\mathrm{M}\right)$, a well-established approach for studying wave propagation in multilayer dielectric structures.

For normal incidence, the characteristic matrix of the $\:\:\mathrm{j}-\mathrm{t}\mathrm{h}$layer is expressed as:^20,23^

1 $$\:{M}_{j}=\left[\begin{array}{cc}\mathrm{cos}\left({\delta\:}_{j}\right)&\:\frac{i}{{n}_{j}}\mathrm{sin}\left({\delta\:}_{j}\right)\\\:i{n}_{j}\mathrm{sin}{\delta\:}_{j}&\:\mathrm{cos}\left({\delta\:}_{j}\right)\end{array}\right]$$

where $\:{n}_{j}$ and $\:{d}_{j}$ are the refractive index and thickness of the layer, respectively, and $\:{\delta\:}_{j}$is the phase thickness given by:

2 $$\:{\delta\:}_{j}=\frac{2\pi\:{n}_{j}{d}_{j}}{{\uplambda\:}}$$

The total transfer matrix of the multilayer structure is obtained by multiplying the individual matrices:

3 $$\:M=\prod\:_{j=1}^{N}{M}_{j}=\left[\begin{array}{cc}A&\:B\\\:C&\:D\end{array}\right]$$

The transmission coefficient is then calculated as:

4 $$\:T=\left|\frac{2{n}_{in}}{{n}_{in}A+\:{n}_{in}{n}_{out}B+\:C+\:{n}_{out}D}\right|2.\frac{{n}_{out}}{{n}_{in}}$$

The introduction of the defect layer breaks the periodicity of the structure, leading to the formation of a localized resonance mode within the photonic band gap. The resonance wavelength is highly sensitive to changes in the refractive index of the defect layer, which forms the basis of the sensing mechanism.

### Performance parameters

The sensor performance is evaluated using the following parameters^19–25^:

Sensitivity:

5 $$\:S=\frac{\varDelta\:\:{\uplambda\:}}{\varDelta\:n}$$

where $\:\varDelta\:n$ is the refractive index variation.

Full Width at Half Maximum (FWHM):

6 $$\:FWHM\:={\:{\uplambda\:}}_{right}-{\:{\uplambda\:}}_{left}$$

Quality factor:

7 $$\:Q=\frac{{{\uplambda\:}}_{\mathrm{r}\mathrm{e}\mathrm{s}}}{FWHM}$$

where $\:{{\uplambda\:}}_{\mathrm{r}\mathrm{e}\mathrm{s}}$ is the resonance wavelength.

Figure of Merit:

8 $$\:FOM=\frac{S}{\:\:FWHM}$$

Detection Accuracy:

9 $$\:DA=\frac{1}{\:\:FWHM}$$

Limit of Detection:

10 $$\:LOD=\frac{\varDelta\:{{\uplambda\:}}_{min}}{S}$$

### Numerical simulation

The numerical analysis is performed using $\:\mathrm{M}\mathrm{A}\mathrm{T}\mathrm{L}\mathrm{A}\mathrm{B}$ based on the transfer matrix method. The structure is defined as:

11 $$\:Air/\left(Ti{O}_{2}/Mg{F}_{2}\right)N/Defect\:\left(Urine\right)/\left(Ti{O}_{2}/Mg{F}_{2}\right)N/Air$$

where the number of periods is $\:N=8$, with layer thicknesses of $\:60\:nm$ for $\:Ti{O}_{2}$ and $\:110\:nm$ for $\:Mg{F}_{2}$, while the defect layer thickness is $\:1500\:nm$ .

## Results and discussion

To strengthen the manuscript in response to the reviewers’ comments, part of the numerical analysis was revisited and refined. In particular, the resonance extraction procedure was re-evaluated to ensure more stable linewidth determination, the refractive-index sensing range was extended up to 1.360, and a fabrication-tolerance analysis was added to examine the effect of moderate thickness deviations around the nominal design. In addition, a comparison with previously reported photonic-crystal-based sensing platforms was included to better position the present work within the existing literature.

**Fig. 1:**
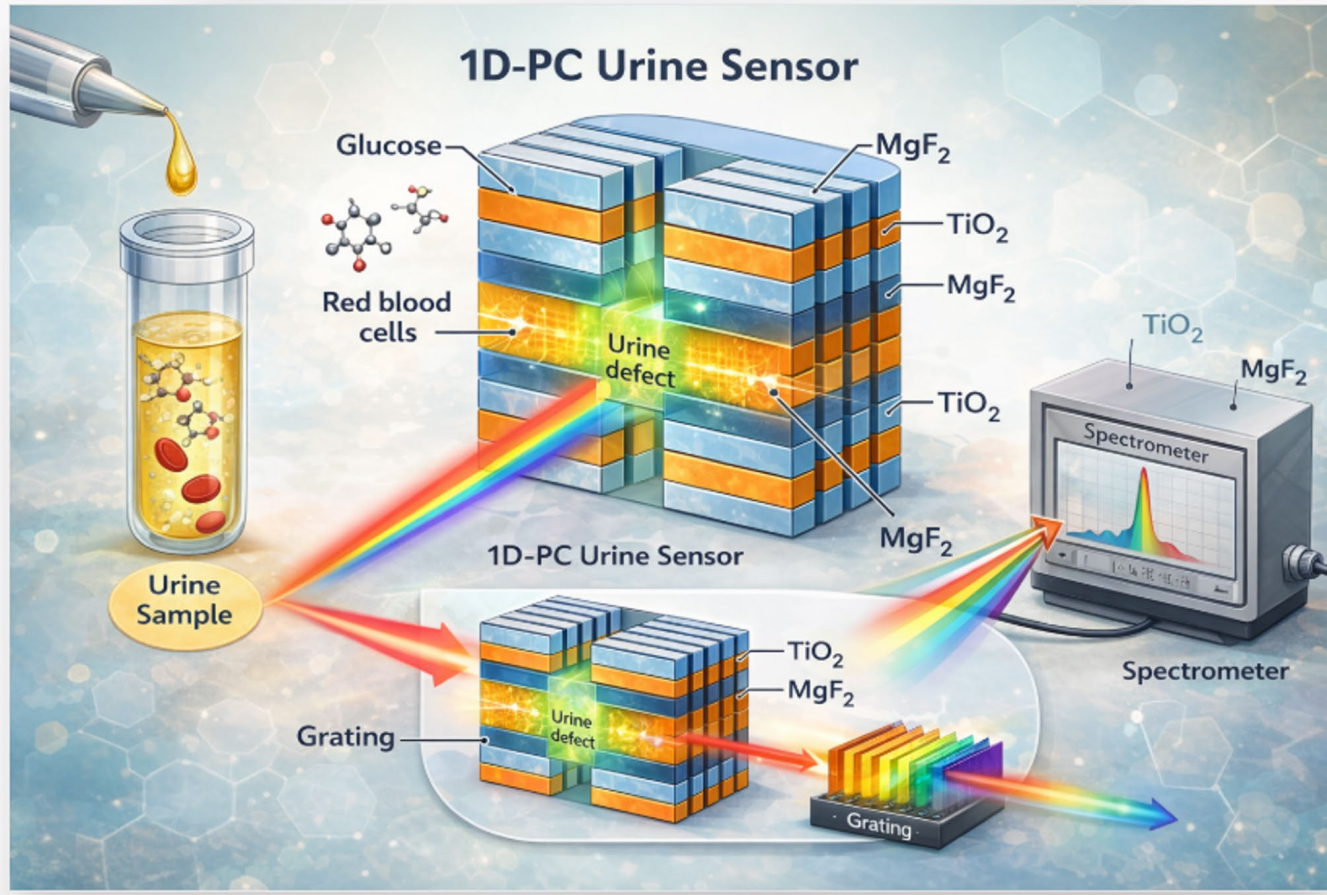
Schematic of the proposed 1D photonic crystal sensor.

For the sensing analysis, the refractive index of the urine defect layer was scanned from 1.333 to 1.360 in order to cover a practically relevant refractometric interval for urine samples. The overall configuration of the proposed sensor is illustrated in Fig. 1, where the TiO₂/MgF₂ Bragg mirrors and the central urine-filled defect cavity are clearly shown. This structure forms the physical basis of the sensing mechanism by enabling strong optical confinement inside the analyte region.

**Fig. 2:**
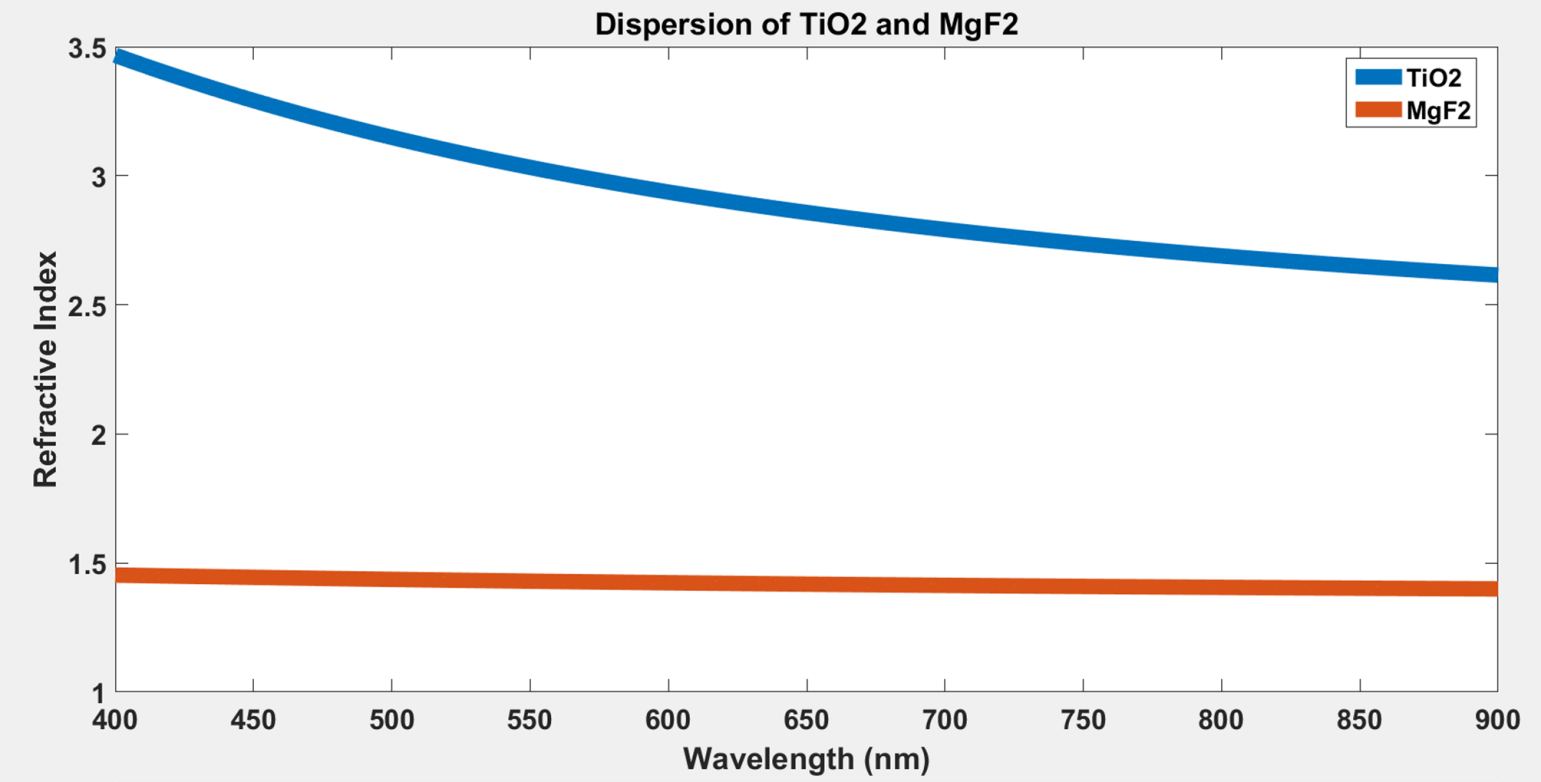
Dispersion of TiO₂ and MgF₂.

The refractive-index dispersion of the constitutive materials is presented in Fig. 2, showing that the contrast between TiO₂ and MgF₂ remains sufficiently large over the investigated spectral range to support strong Bragg reflection and stable band-gap formation.

**Fig. 3:**
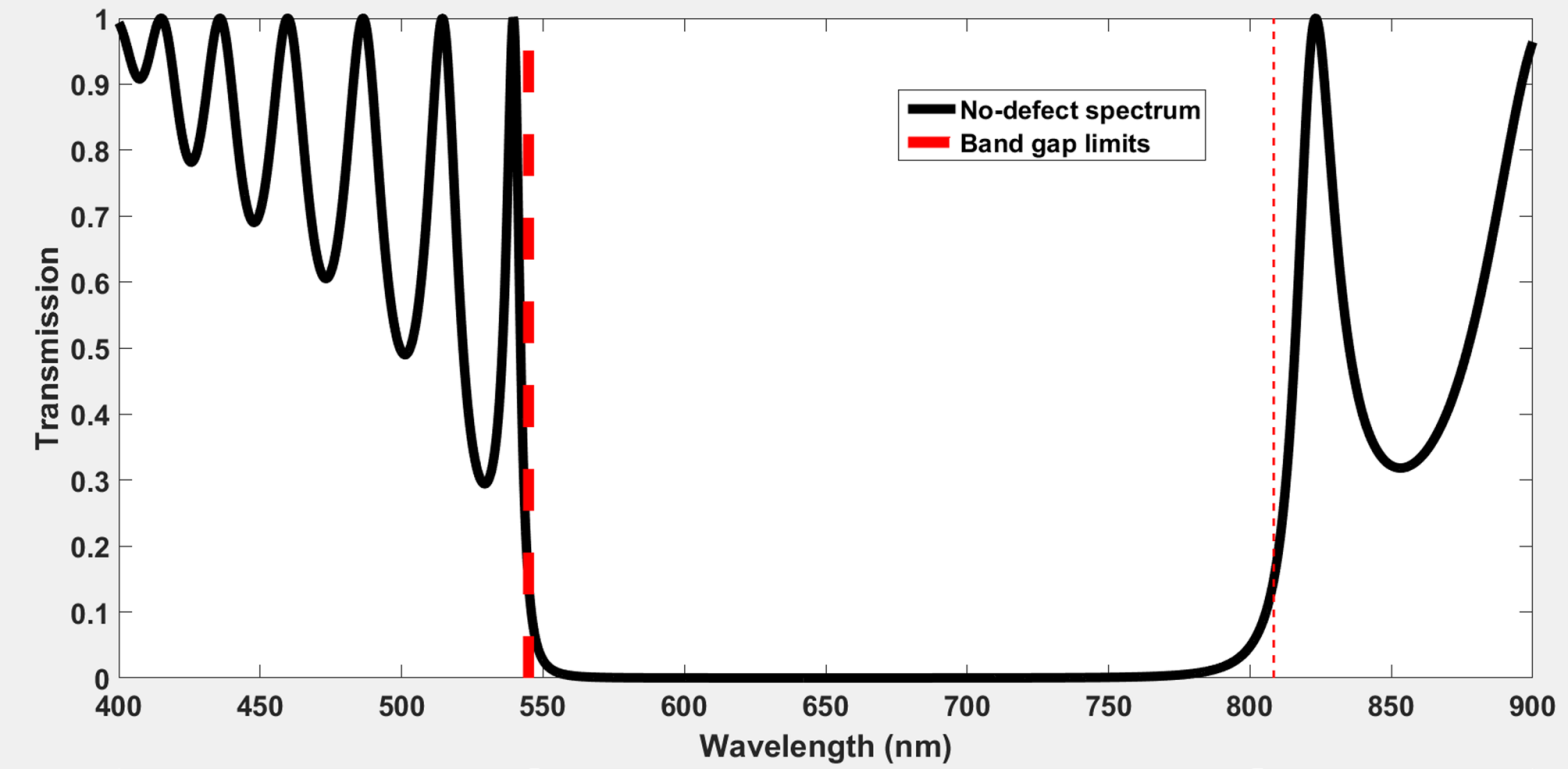
Defect-free spectrum showing the photonic band gap.

Based on this refractive-index contrast, the defect-free multilayer exhibits a clear photonic band gap, as shown in Fig. 3. The appearance of this forbidden band confirms that the periodic TiO₂/MgF₂ arrangement provides the required spectral confinement for hosting localized defect modes.

**Fig. 4:**
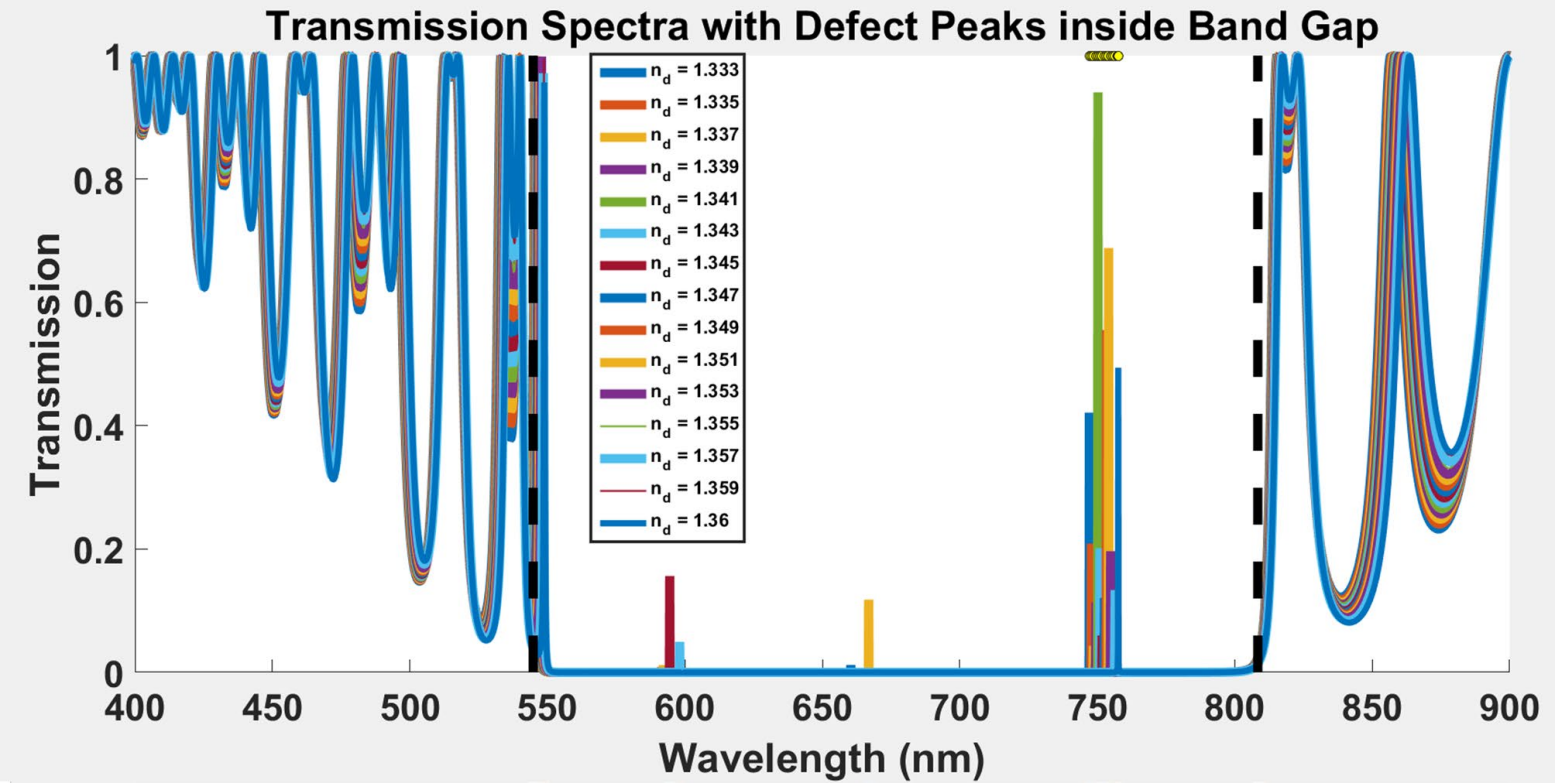
Transmission spectra with defect resonances inside the band gap.

**Fig. 5:**
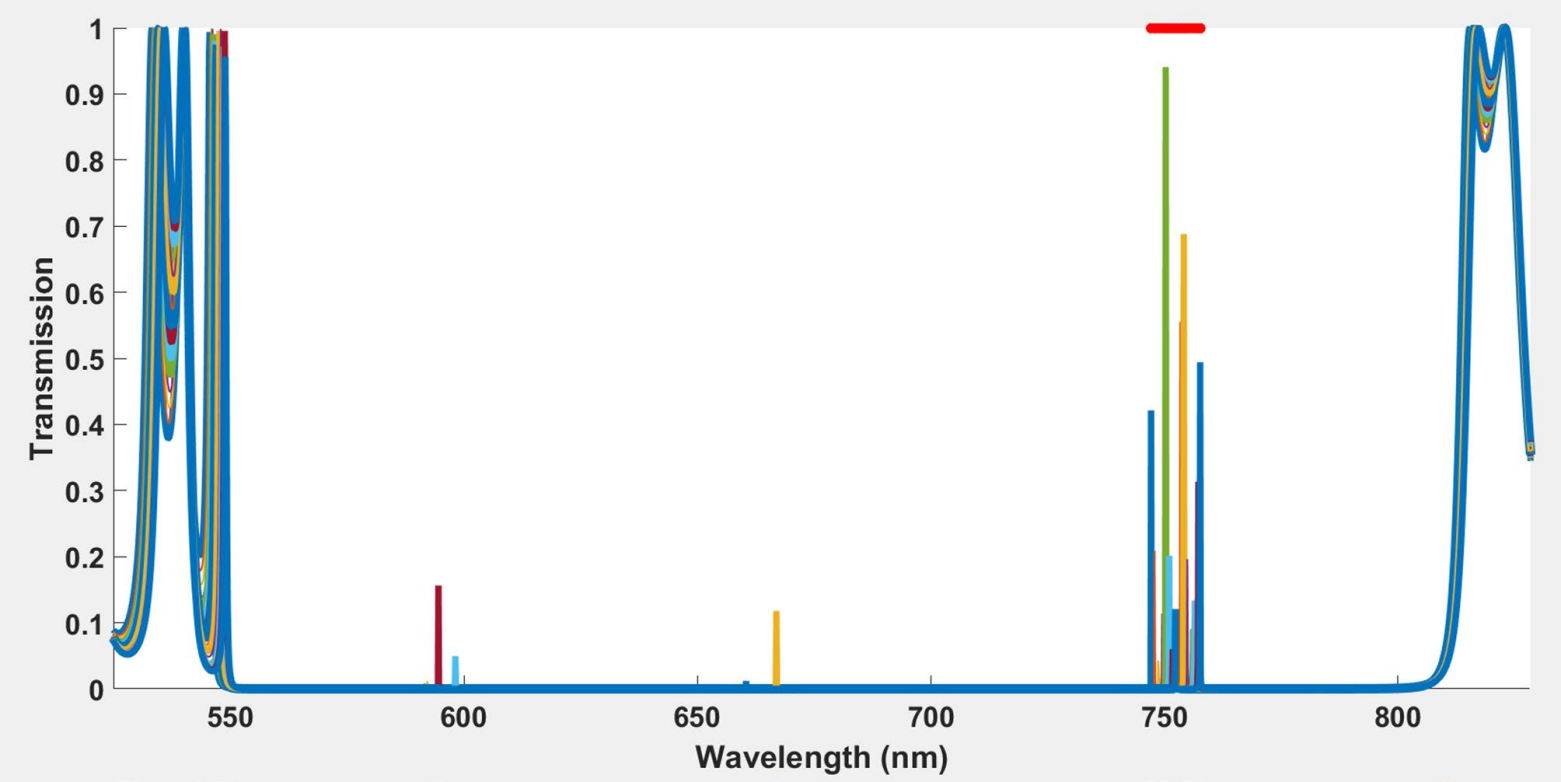
Zoomed view of the defect resonance.

After introducing the urine-filled cavity, localized resonances emerge inside the band gap, as demonstrated in Fig. 4. A magnified view of the dominant resonance is provided in Fig. 5, where the defect mode appears sharp and well resolved, confirming the successful formation of a localized optical state due to the intentional breaking of structural periodicity.

**Fig. 6:**
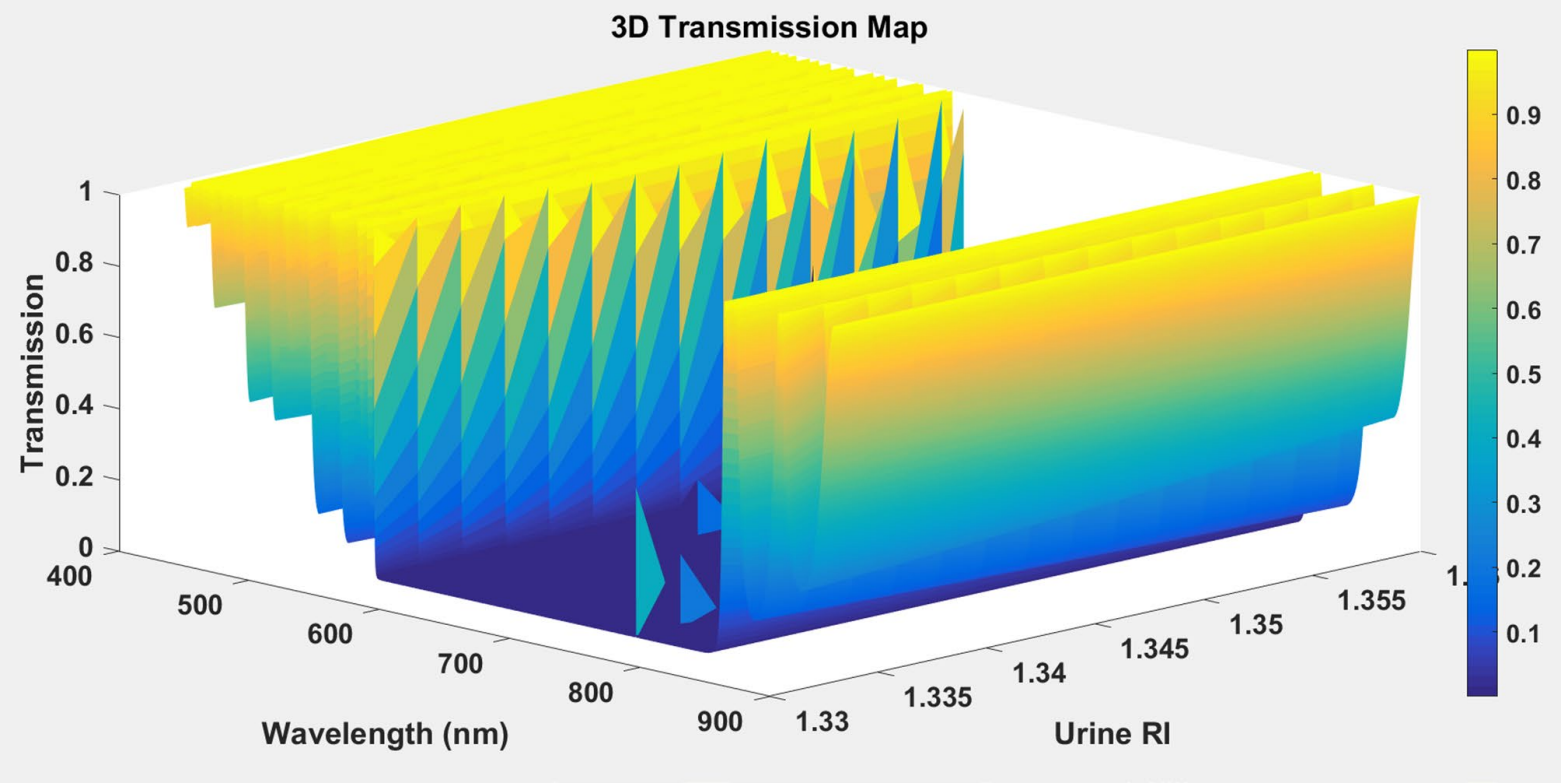
The 3D transmission map versus wavelength and urine RI.

**Fig. 7:**
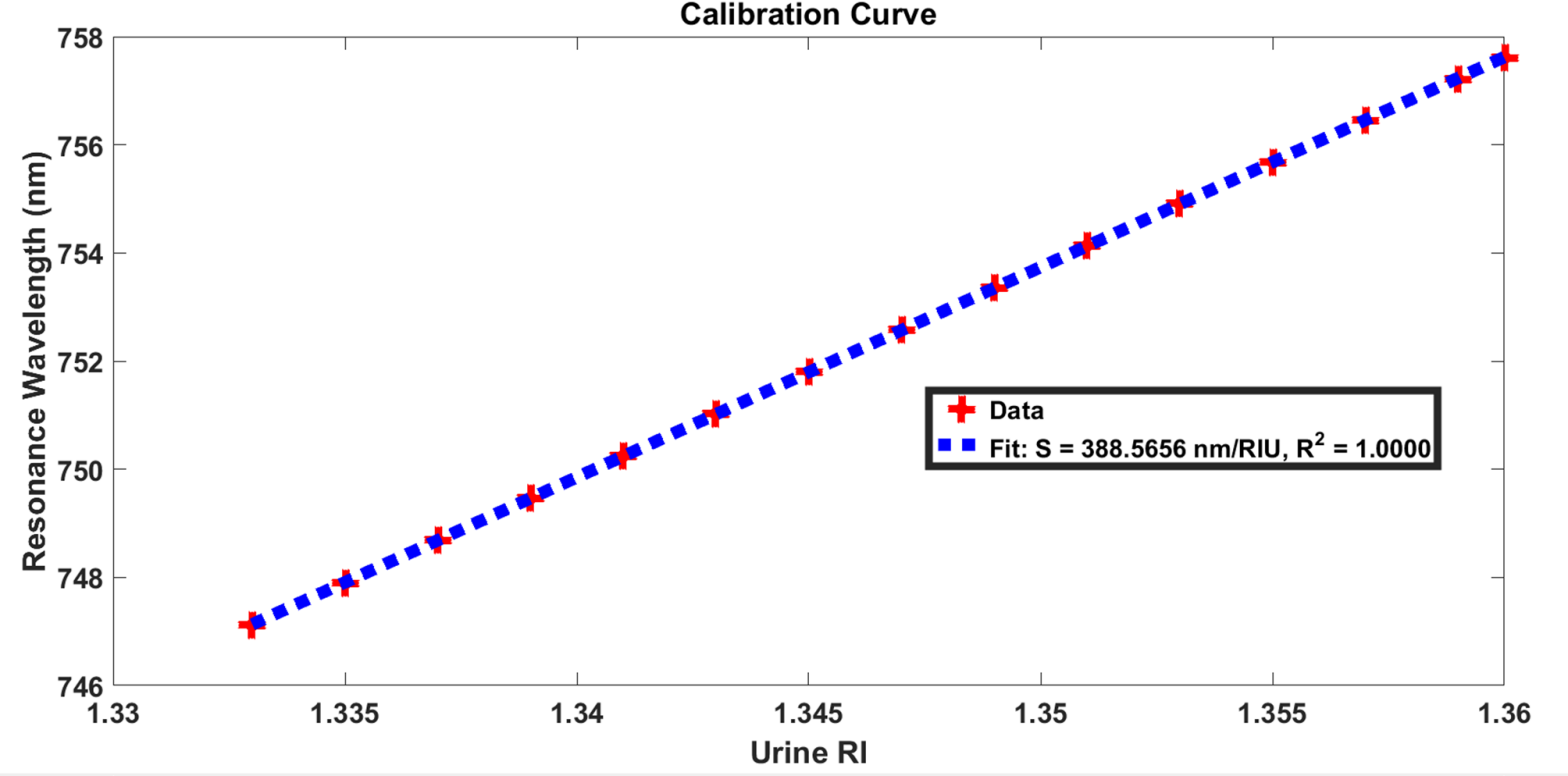
Calibration curve of resonance wavelength versus urine RI.

The evolution of this defect resonance with urine refractive index is illustrated in Fig. 6, where the 3D transmission map shows a continuous red shift as the refractive index increases. This monotonic behavior is quantified in Fig. 7, which presents the calibration curve between the resonance wavelength and the urine refractive index. After re-evaluation of the numerical extraction procedure and extension of the sensing interval up to $\:n=1.360$, the proposed structure achieves an updated sensitivity of 388.57 nm/RIU with an excellent linearity of $\:{R}^{2}=0.999989$. The resonance wavelength shifts from 747.1120 nm at $\:n=1.333\:to\:757.6045nm$ at $\:n=1.360$, confirming that the sensor remains stable over the extended operating range. This behavior reflects the increase in the effective optical thickness of the defect cavity as the urine refractive index increases, which alters the resonance condition in a predictable and continuous way.

**Fig. 8:**
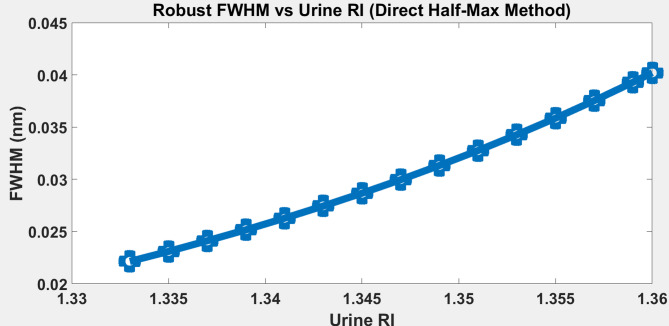
FWHM variation with urine RI.

The spectral sharpness of the device is examined through the linewidth behavior shown in Fig. 8 (Robust FWHM vs. Urine RI). The full width at half maximum increases gradually from 0.0221 nm to 0.0402 nm across the investigated refractive-index range, with an average value of 0.0305 nm.

**Fig. 9:**
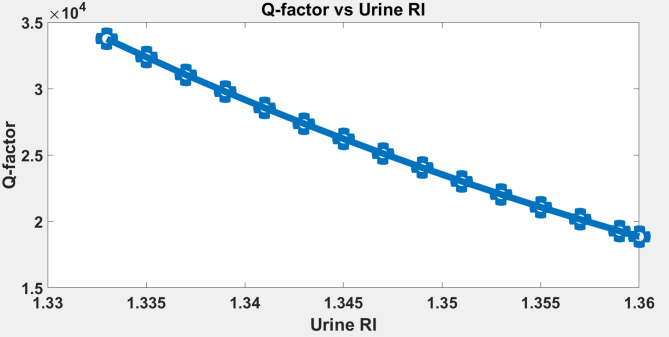
Q-factor variation with urine RI.

Although a moderate increase in linewidth is expected as the resonance moves across the band gap, the mode remains extremely narrow throughout the entire sensing range. This narrow linewidth is a central strength of the proposed design because it directly improves spectral resolution and enhances the reliability of resonance tracking. The inverse relation between linewidth and resonance quality is confirmed in Fig. 9 (Q-factor vs. Urine RI). As the FWHM increases, the Q-factor decreases gradually from $\:3.37\times\:{10}^{4}\:to\:1.88\times\:{10}^{4}$, with an average value of $\:2.55\times\:{10}^{4}.\:$.

**Fig. 10:**
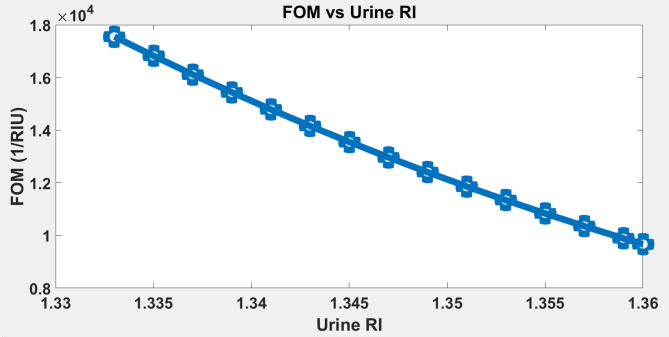
FOM variation with urine RI.

Despite this reduction, the Q-factor remains on the order of $\:{10}^{4}$, which indicates that the resonance retains excellent confinement and high spectral selectivity across the full sensing interval. This trend is also reflected in Fig. 10 (FOM vs. Urine RI), where the figure of merit remains high, yielding an average value of $\:1.32\:\times\:{10}^{4}RI{U}^{-1}$. The simultaneous presence of high sensitivity and narrow linewidth confirms that the sensing performance of the proposed structure is not based on resonance shift alone, but rather on a favorable balance between wavelength sensitivity, spectral sharpness, and resonance quality.

**Fig. 11:**
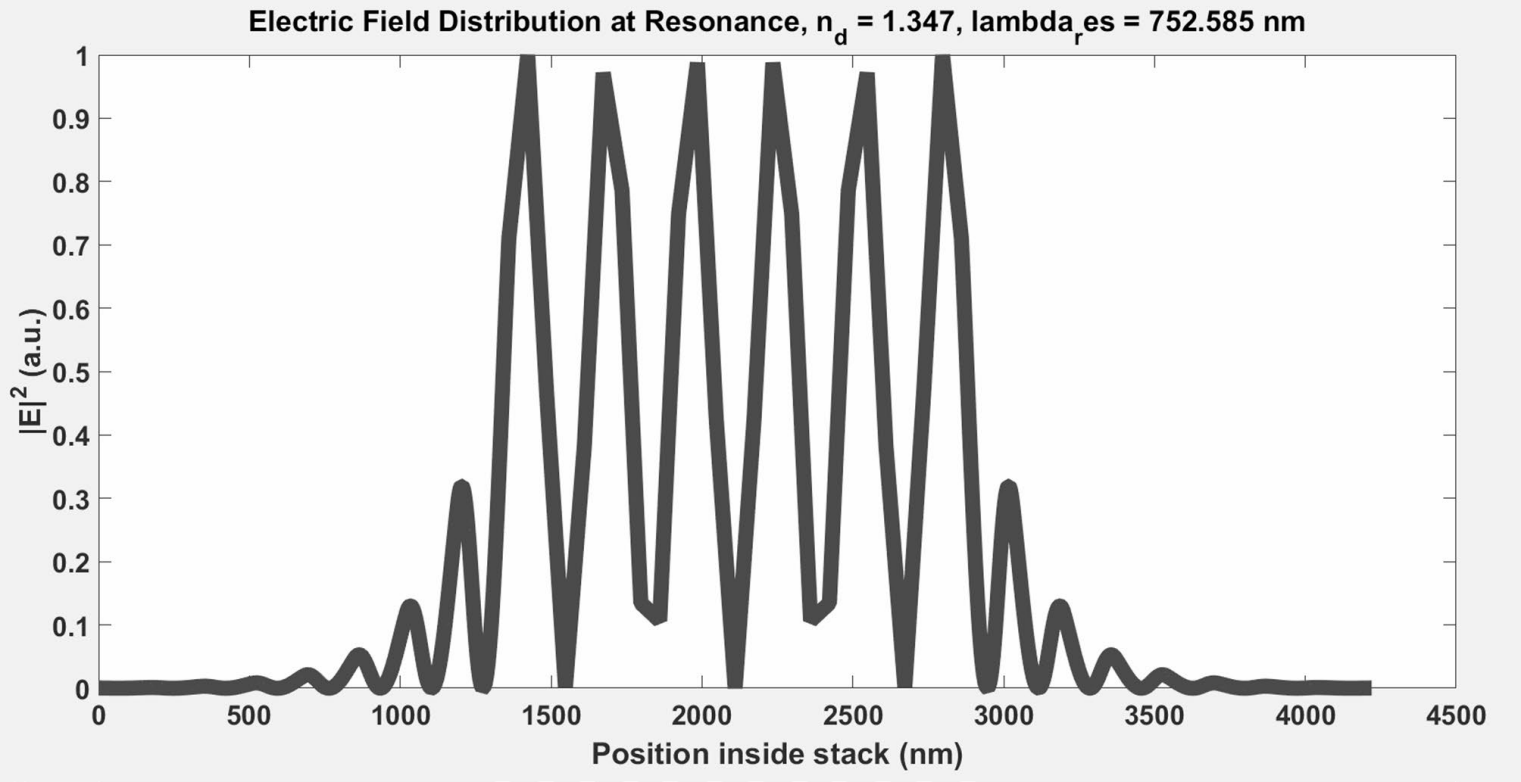
Electric-field distribution at resonance.

Further physical insight is provided by Fig. 11, which presents the electric-field distribution at resonance. The field is strongly localized inside the urine defect cavity, confirming that the analyte region acts as the main interaction zone between the optical mode and the sample. This strong field confinement explains the pronounced sensitivity of the resonance wavelength to refractive-index variation.

**Fig. 12:**
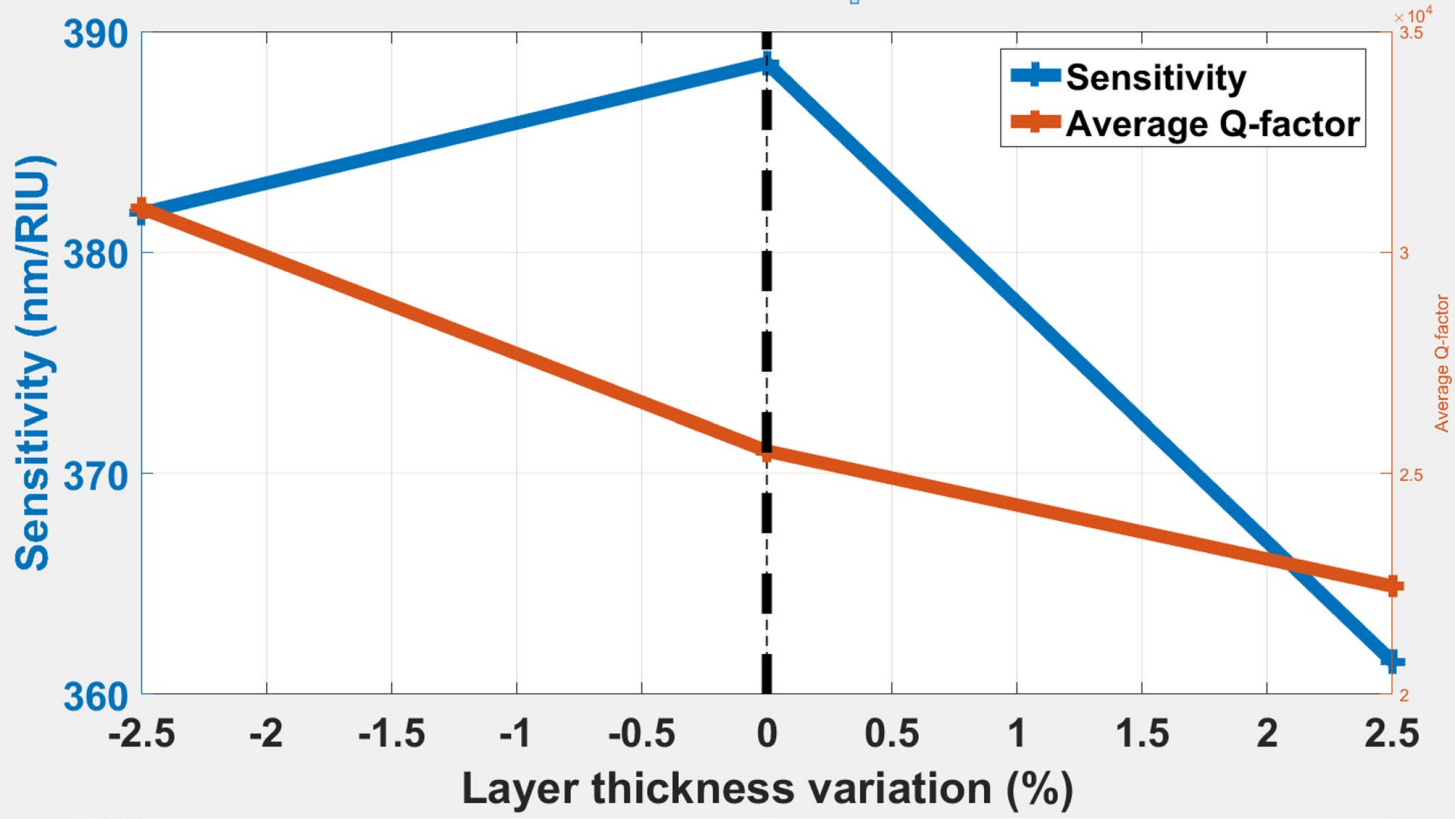
Tolerance analysis of sensitivity and average Q-factor under ± 2.5% thickness variation.

**Fig. 13:**
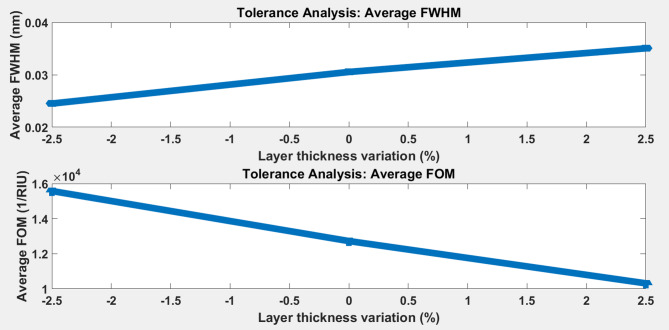
Tolerance analysis of average FWHM and average FOM.

To assess the fabrication robustness of the proposed sensor, a thickness-tolerance analysis was carried out by introducing moderate deviations of − 2.5%, 0%, and + 2.5% around the nominal layer thicknesses. The selected layer thicknesses (60 nm for TiO₂, 110 nm for MgF₂, and 1500 nm for the defect cavity) are within the practical fabrication range of current dielectric thin-film deposition techniques, and the additional ± 2.5% tolerance analysis further confirms that the proposed sensor can maintain stable optical performance under realistic dimensional deviations. The corresponding results are presented in Figs. 12 and 13. Figure 12 shows that the sensitivity remains high for all investigated cases, ranging from 381.77 nm/RIU at − 2.5% deviation to 388.57 nm/RIU for the nominal structure and 361.48 nm/RIU at + 2.5% deviations. In parallel, the average Q-factor remains on the order of $\:{10}^{4}$, decreasing from $\:3.10\times\:{10}^{4}\:to\:2.24\:\times\:{10}^{2}$. As shown in Fig. 13, the average FWHM increases gradually from 0.0245 nm to 0.0351 nm, accompanied by a corresponding decrease in the average FOM. Despite these changes, the resonance remains narrow and well defined, confirming that the proposed design possesses good tolerance against moderate fabrication variations.

A key outcome of the revised analysis is that the updated calculations provide a more reliable and physically consistent description of the sensor performance than the earlier version of the manuscript. The refined resonance extraction yields more stable linewidth determination and significantly improved Q-factor and FOM values. Thus, the revised results demonstrate that the present sensor is not characterized by sensitivity alone, but rather by a favorable combination of high sensitivity, narrow linewidth, strong field localization, high resonance quality, and good fabrication tolerance.

Temperature-induced resonance shifts are physically expected because of the thermo-optic response of both the constituent materials and the analyte; however, a detailed investigation of thermal cross-sensitivity is beyond the scope of the present study and will be addressed in future work.

**Table 1:** Comparison of the sensitivity of the proposed urine-based 1D photonic crystal sensor with representative previously reported photonic-crystal sensing structures. [Note that some reported sensitivities were originally expressed based on angular interrogation.]

Sample details	Q	FWHM	Sensitivity	Structure
^26^	NR	NR	~ 200 °/RIU	Graphene–MoS₂ hybrid
^27^	63.51	3.70 (deg.)	~ 235 °/RIU	WS2 monolayer
^28^	87.875	33.3969 RIU⁻¹	267 nm RIU⁻	Crystalline GST phase
	82.5882	28.8568 RIU⁻¹	245 nm RIU⁻¹	amorphous GST phase
^29^	605	59.5 RIU⁻¹	208 nm/RIU	GaN-based PC
^30^	36.16	405.50 RIU⁻¹	261.33 °/RIU	MoS₂-based biosensor
^31^	NR	NR	35 (nm/RIU)	2D L^3^ PhC
^32^	NR	NR	240 (nm/RIU)	PC waveguide
^33^	NR	NR	200 (nm/RIU)	PC slab waveguide
^34^	NR	NR	260 (nm/RIU)	PC waveguide
^35^	100.59 RIU⁻¹	100.59 RIU⁻¹	290.714 deg/RIU	Blood cancer (Jurkat)
Presented work	$\:2.55\times\:{10}^{4}$	$\:1.32\:\times\:{10}^{2}RI{U}^{-1}$	381.77 ~ 388.57 nm/RIU	Urine 1DPhCs

Table 1 shows that the proposed urine-based 1D photonic crystal sensor achieves a sensitivity in the range of 381.77–388.57 nm/RIU, together with a high Q-factor of 2.55 × 10^4^ and a large FOM of 1.32 × 10^4^ RIU^− 1^. When compared with representative previously reported photonic sensing structures, including graphene–MoS₂ hybrid, WS₂ monolayer, GST-based, GaN-based, MoS₂-based, 2D L3 photonic crystal, and photonic-crystal waveguide platforms, the present design demonstrates a reasonably strong and well-balanced sensing performance. Although some published studies have reported higher sensitivity values, the purpose of this comparison is not to claim the highest sensitivity alone. In optical and photonic sensing, the practical performance of a sensor should be evaluated through the combined interplay of sensitivity, spectral linewidth, Q-factor, figure of merit, resonance stability, and fabrication tolerance. From this perspective, Table 1 is provided mainly for guidance and contextualization, in order to indicate the relative position of the proposed sensor among previously reported platforms. Therefore, the significance of the present design lies not only in achieving a competitive sensitivity, but also in offering a favorable overall spectral performance supported by narrow linewidth, high resonance quality, strong defect-mode confinement, and acceptable robustness against fabrication variations.

Compared with two-dimensional and three-dimensional photonic crystal configurations, the proposed one-dimensional architecture offers several practical advantages, including simpler fabrication, easier structural optimization, and straightforward resonance analysis through the transfer matrix method. While higher-dimensional photonic crystals may provide stronger lateral confinement or compact cavity geometries, they are often associated with greater fabrication complexity and stricter tolerance requirements. In contrast, the present 1D defect-engineered structure can generate a sharp localized resonance with very narrow linewidth, high Q-factor, and stable spectral behavior using a comparatively simple multilayer configuration. For this reason, the 1D platform was selected in this work as a practical and efficient architecture for urine refractive-index sensing.

## Conclusion

In this work, a defect-engineered one-dimensional photonic crystal sensor based on alternating TiO₂/MgF₂ layers with a urine-filled central cavity was proposed and numerically investigated for urine refractive-index detection. The results confirmed the formation of a clear photonic band gap and a sharp localized defect mode that shifts systematically with changes in the urine refractive index. Over the extended sensing range from 1.333 to 1.360, the proposed sensor achieved a high sensitivity of 388.57 nm/RIU with excellent linearity$\:({R}^{2}=0.999989)$. In addition, the structure maintained a very narrow average linewidth of 0.0305 nm, a high average Q-factor of $\:2.55\times\:{10}^{4}$,, and a large average FOM of $\:1.32\times\:{10}^{2}RI{U}^{-1}$, reflecting strong spectral selectivity and high sensing quality. The electric-field analysis further demonstrated strong confinement inside the defect cavity, confirming enhanced light–matter interaction in the analyte region. A thickness-tolerance analysis under moderate deviations of − 2.5%, 0%, and + 2.5% also showed that the sensor preserves stable performance under fabrication variations. These findings indicate that the proposed design offers not only high sensitivity, but also narrow linewidth, high resonance quality, and good structural robustness. Therefore, the present urine-based 1D photonic crystal sensor represents a promising platform for high-resolution biomedical refractive-index sensing. Comparison with representative previously reported photonic sensing platforms further supports that the value of the proposed design lies not only in its reasonable sensitivity, but also in its balanced spectral performance and structural robustness.

## Data Availability

The datasets used and analyzed in this study are available upon reasonable request from the corresponding author.

## References

[1] Ochoa, M. al. Recent Advances in Biomedical Photonic Sensors: A Focus on Optical-Fibre-Based Sensing. *Sensors* **21**(19), 6469. 10.3390/s21196469 (2021). 34640788 10.3390/s21196469 PMC8513032

[2] Fathi, F. et al. Photonic crystal based biosensors: Emerging inverse opals for biomarker detection. *Talanta* **221**, 121615. 10.1016/j.talanta.2020.121615 (2021). 33076145 10.1016/j.talanta.2020.121615 PMC7466948

[3] Panda, A. et al. Graphene-based 1D defective photonic crystal biosensor for real-time detection of cancer cells. *Eur. Phys. J. Plus*. **136**, 809. 10.1140/epjp/s13360-021-01796-z (2021).

[4] Abohassan, K. M. et al. 1D photonic crystal-based sensor for detection of cancerous blood cells. *Opt. Quant. Electron.* **53**, 1. 10.1007/s11082-021-03014-7 (2021).

[5] Mohamed, B. A. et al. Theoretical investigation of fast illicit drug detection via ternary photonic crystals. *Sci. Rep.* **16**, 11240. 10.1038/s41598-026-39408-4 (2026). 41927640 10.1038/s41598-026-39408-4 PMC13046969

[6] Nouman, W. M. et al. Chemical-enhanced thyroid cell detection using photonic crystal biosensors with phase-change materials. *RSC Adv.* **16**, 10113–10128 (2026). 41726223 10.1039/d5ra09237j PMC12917951

[7] Ayyanar, N. et al. Photonic crystal fiber-based refractive index sensor for early detection of cancer. *IEEE Sens. J.* **18**, 7093–7099. 10.1109/JSEN.2018.2854375 (2018).

[8] Ouardi, M. E. et al. Multi-purpose Surface Plasmon Resonance Sensor with Enhanced Sensitivity for Detecting Anemia and Monitoring Glucose Levels. *Plasmonics* **20**, 8253–8266. 10.1007/s11468-025-02813-y (2025).

[9] Zhang, Y. et al. Protein sensing using deep subwavelength-engineered photonic crystals. *Opt. Lett.* **49** (2), 395–398. 10.1364/OL.510541 (2024). 38194577 10.1364/OL.510541

[10] Balaji, V. R. et al. Machine learning enabled 2D photonic crystal biosensor for early cancer detection. *Measurement* **224**, 113858. 10.1016/j.measurement.2023.113858 (2024).

[11] Hamed, M. M. et al. Compact 2-D photonic crystal biomedical sensor for enhanced glucose concentration detection in urine. *Sci. Rep.* **15**, 4905. 10.1038/s41598-025-87547-x (2025). 39929943 10.1038/s41598-025-87547-x PMC11811212

[12] Maher, M. A. et al. Maximizing temperature sensitivity in a one-dimensional photonic crystal thermal sensor. *Sci. Rep.* **15**, 4105. 10.1038/s41598-024-82889-4 (2025). 39901016 10.1038/s41598-024-82889-4 PMC11791204

[13] Alshomrany, A. S. et al. Vitiligo detection capabilities of 1D photonic crystal biosensing design. *Sci. Rep.* **15**, 883. 10.1038/s41598-024-83421-4 (2025). 39762338 10.1038/s41598-024-83421-4 PMC11704009

[14] Barvestani, J. Topological interface state-based photonic crystal sensor with porous cap layer for high-performance biosensing. *Sci. Rep.* **15**, 43675. 10.1038/s41598-025-27526-4 (2025). 41387751 10.1038/s41598-025-27526-4 PMC12700879

[15] Mostufa, S. et al. Advancements and Perspectives in Optical Biosensors. *ACS Omega*. **9**(23), 24181–24202. 10.1021/acsomega.4c01872 (2024). 38882113 10.1021/acsomega.4c01872 PMC11170745

[16] Ouardi, M. E. et al. Development of a Novel SPR Biosensor for Early Pregnancy Detection. *Sens. Imaging*. **26**, 56. 10.1007/s11220-025-00582-w (2025).

[17] Ouardi, M. E. et al. Detection of Water-alcohol Content Using Surface Plasmon Resonance. *Plasmonics* **20**, 399–406. 10.1007/s11468-024-02285-6 (2025).

[18] Vaz, R. et al. Green photonic biosensing: Approaching sustainability in point-of-care diagnostics. *TRAC Trends Anal. Chem.* **177**, 117771. 10.1016/j.trac.2024.117771 (2024).

[19] Ramola, A. et al. Recent Advances in Photonic Crystal Fiber-Based SPR Biosensors: Design Strategies, Plasmonic Materials, and Applications. *Micromachines* **16**(7), 747. 10.3390/mi16070747 (2025). 40731657 10.3390/mi16070747 PMC12298052

[20] Aly, A. H. et al. CaF2/TiO2 nanophotonic biosensor based on a one-dimensional photonic crystal for Chikungunya virus detection. *Appl. Opt.* **63**(30), 7909–7916. 10.1364/AO.537230 (2024).

[21] Hu, H. et al. High-Sensitivity Terahertz Biosensor Based on a Multi-Layer Hybrid Structure Consisting of a Defect Mode and Graphene. *Biosensors* **15**, 702. 10.3390/bios15100702 (2025). 41149354 10.3390/bios15100702 PMC12564660

[22] Dewal, S. et al. Refractive index engineering in periodic and quasi-periodic 1d photonic crystals for improved optical sensing. *J. Opt.*10.1007/s12596-026-03105-2 (2026).

[23] Yeh, P. *Optical Waves in Layered Media* (Wiley, 1988).

[24] Zaghdoudi, J., Giden, I. H. & Kanzari, M. Next-generation 1D photonic crystal sensor: revolutionizing fat concentration measurement in commercial milk. *Phys. B Condens. Matter*. **714**, 417428 (2025).

[25] Singh, M. K. et al. Optical biosensors: a decade in review. *Alexandria Eng. J.* **67**, 673–691. 10.1016/j.aej.2022.12.040 (2023).

[26] Vahed, H. et al. Sensitivity enhancement of SPR optical biosensor based on Graphene–MoS₂ structure with nanocomposite layer. *Opt. Mater.* **88**, 161–166. 10.1016/j.optmat.2018.11.034 (2019).

[27] Kumar, A., Yadav, A. K., Kushwaha, A. S. & Srivastava, S. K. A comparative study among WS₂, MoS₂ and graphene based surface plasmon resonance (SPR) sensor. *Sens. Actuators Rep.* **2**(1), 100015. 10.1016/j.snr.2020.100015 (2020).

[28] Walaa, M. et al. Chemical-enhanced thyroid cell detection using photonic crystal biosensors with phase-change materials. *RSC Adv.* **16**, 10113–10128. 10.1039/D5RA09237J (2026). 41726223 10.1039/d5ra09237j PMC12917951

[29] N. H. and A. S., Design and analysis of a GaN-based 2D photonic crystal biosensor integrated with machine learning techniques for detection of skin diseases. *Sci. Rep.* **15**, 41863. 10.1038/s41598-025-25893-6 (2025). 10.1038/s41598-025-25893-6 PMC12647168 41290818

[30] Tene, T., Tubon-Usca, G., Gallegos, K. T., Mendoza Salazar, M. J. & Vacacela Gomez, C. MoS₂-based biosensor for SARS-CoV-2 detection: A numerical approach. *Front. Nanotechnol.* **6**, 1505751. 10.3389/fnano.2024.1505751 (2025).

[31] Zhang, L. et al. Yan Study of photonic crystal cavity sensor integrated with microfluidic channel in the visible region. *Proc. SPIE 8561, Advanced Sensor Systems and Applications V* (2013). 10.1117/12.999548

[32] Bougriou, F., Bouchemat, T., Bouchemat, M. & Paraire, N. High sensitivity of sensors based on two-dimensional photonic crystal. In *Proceedings of the 2011 Saudi International Electronics, Communications and Photonics Conference (SIECPC)* 1–4 (2011).

[33] Singh, S., Sinha, R. K. & Bhattacharyya, R. Photonic crystal slab waveguide-based infiltrated liquid sensors: Design and analysis. *J. Nanophotonics*. **5**, 053505 (2011).

[34] Dutta, H. S. & Pal, S. Design of a highly sensitive photonic crystal waveguide platform for refractive index based biosensing. *Opt. Quantum Electron.* **45**, 907–917 (2013).

[35] Vikash, K. et al. Highly sensitive Ag/BaTiO3/MoS2 nano composite layer based SPR sensor for detection of blood and cervical cancer. *Results Opt.* **14**,100597. 10.1016/j.rio.2023.100597 (2024).

